# The Characteristics of Virtual Child Sexual Abuse Material Offenders and the Harms of Offending: A Qualitative Content Analysis of Print Media

**DOI:** 10.1007/s12119-023-10091-1

**Published:** 2023-05-31

**Authors:** Larissa S. Christensen, Noah Vickery

**Affiliations:** 1 Sexual Violence Research and Prevention Unit, School of Law and Society, University of the Sunshine Coast, 90 Sippy Downs Drive, Maroochydore, DC, QLD 4558 Australia; 2 Law and Society Student, School of Law and Society, University of the Sunshine Coast, 90 Sippy Downs Drive, Maroochydore, DC, QLD 4558 Australia

**Keywords:** Child sexual abuse, Virtual, Cartoon, Child abuse material, Child sex offender, Media

## Abstract

Child sexual abuse material (CSAM) has become a global problem. With technological advancements, a sub-type of material has emerged: virtual child sexual abuse material (VCSAM). Despite the far-reaching harms of this material, little is known about VCSAM offenders. Research has found some disconnect between the perceived harmfulness of VCSAM and legislative definitions, compared with CSAM. Given the media’s influential ability to shape public perceptions, this exploratory study aimed to: (1) identify the characteristics of VCSAM offenders and (2) explore whether the harms of VCSAM are represented in this reporting. For the most up-to-date data, the search spanned 1 January 2019 to 23 September 2022. Of the 160 newspaper articles that appeared, 25 met the inclusion criteria. Four themes emerged from the qualitative content analysis: (1) VCSAM is a form of CSAM, (2) potential for escalation in offending from viewing VCSAM, (3) offenders have preferences in specific types of VCSAM (with the sub-theme: written stories and documents are VCSAM too), and (4) offenders “didn’t know” the material was illegal. Overall, these findings were positive; how cases were reported may broadly educate the public about VCSAM offending, with articles signifying the harms of this offending. The current findings have the potential to contribute to prevention and intervention efforts, with utility in policymaking, criminal justice, media, and psychology disciplines.

## Introduction

Child sexual abuse material (CSAM) presents significant challenges to law enforcement. Cooper’s (1998) concept of the Triple-A Engine has long posited that the ease of accessibility, affordability, and anonymity of the internet has streamlined variances among material and perpetrators. There has been an explosion of CSAM in recent years; the National Center for Missing and Exploited Children (NCMEC) in the United States recorded 23.4 million CSAM cases between 1998 and 2017, with 40% being recorded in 2017 alone (Bursztein et al. 2019). The COVID-19 pandemic has since proliferated CSAM figures due to various factors, including social isolation and increased time spent online (Europol 2020). Evolving technologies foster novel opportunities for CSAM offenders (Seto, 2013). With enhancements in technology, one area policymakers and law enforcement are now contending with is the emerging threat of virtual child sexual abuse material (VCSAM) (Christensen et al., 2021). However, very little is known in the literature about VCSAM offenders.

### Literature Review

VCSAM is a sub-type of CSAM and is defined through a number of variants. It can include entirely computer-generated images, altering an image of a child to look like they are engaging in sexual activity, or altering a sexualized image of an adult to look like a child (Avery, 2015; Warner, 2010). VCSAM exists in various formats including cartoons, drawings, animations, literature, recorded audio, and sculptures (Christensen et al., 2021). While fictitious children are often the subjects in VCSAM, alarmingly, offenders can use software to manipulate images and recordings of *real* child victims to appear fictional (e.g., making the image appear cartoon-like), disguising the abuse of a real victim (Northern Ireland Office, 2007). Acknowledging that legislation differs across countries, the law has evolved in response to the emergence of CSAM and criminalizes such content in any form, typically with the inclusion of VCSAM (Moritz et al., 2022). Although, it is important to note that there are challenges regarding legislative definitions, given the speed at which these offenses change (Cullen et al., 2020).

VCSAM has significantly harmful implications. This includes its use to aid in the violation of children’s privacy and extortion, defamation, disguising CSAM, and grooming (Clough, 2012). Recently, debates have emerged about the fictional status of VCSAM, weighing freedom of speech and artistic expression against the consequences of normalizing any depiction of sexual acts against a child (Al-Alosi, 2018; Jung, 2021). Research has found that young adults perceived continually viewing and distributing CSAM to lead to further production and negative effects for victims (Prichard et al. 2015). Maras and Shapiro (2017) argue that VCSAM does not prevent the escalation of pedophilic behavior. Conversely, it can progress CSAM addiction. VCSAM can fuel the abuse of children by legitimizing and reinforcing one’s views of children (Northern Ireland Office, 2007). The material can also be used in the grooming of children, reducing the inhibitions of children, and normalizing and desensitizing the sexual demands (Cohen-Almagor, 2013), particularly if the VCSAM was to depict the victim’s favorite cartoon character engaged in the sexual activity in a conceding and happy way (Christensen et al., 2021).

Despite the harms, research has found inconsistencies concerning public perspectives on the seriousness of CSAM (Warner, 2010). In fact, research has identified some disconnect between legislative definitions of VCSAM and social attitudes (Prichard et al. 2015). Prichard et al. (2015) found that while 7% of participants agreed that CSAM should not be illegal, 22% agreed VCSAM should not be illegal. These findings suggest there is a greater amount of disconnect between the perceived harmfulness of VCSAM and legislative definitions, compared with CSAM. One reason for this finding might be that individuals believe no real victims are involved in the production of VCSAM, failing to recognize the additional harms. As CSAM laws relating to VCSAM are still somewhat new, it has been argued that social perceptions of the legislation may still be evolving and are possibly influenced by public debates (Prichard et al. 2015). It is, therefore, imperative to consider the reporting of VCSAM cases in the media.

The media has the potential to significantly influence society’s understanding of CSAM offenders (Ball-Rokeach & DeFleur, 1976; Christensen, 2018; Jackob, 2010; Tai & Sun, 2007), and thus, including VCSAM offenders. Media outlets currently hold hegemony over the shaping of public perception (Gray, 2013) hence the importance of exploring the messages disseminated to the public. Further to shaping public beliefs along with values and myths, the media can hinder or stimulate policies (Berkeley Media Studies Group 2003). While the media has the capacity to marginalize and obstruct victims through the misconstruction of reporting offenses (Hayes & Baker, 2014; Hetherton, 1999), it can also have a positive influence on preventing crime through the publicization of outcomes such as arrests and sentences (Baron & Kennedy, 1998).

While our knowledge of CSAM offenders is growing, presently, there is a dearth of literature on VCSAM offenders. This is concerning, particularly when child welfare and law enforcement experts have been raising concerns for over a decade that VCSAM is increasing and will continue to do so (Northern Ireland Office, 2007). The clear disconnect between the perceived harmfulness of VCSAM and legislative definitions, compared with CSAM, begs for the exploration of VCSAM reporting in the media. Such research is imperative given the media’s influential ability to shape public perceptions (Gray, 2013) and the harmful implications of VCSAM (Christensen et al., 2021). The aim of this exploratory study is twofold and sought to: (1) identify the characteristics of VCSAM offenders and (2) explore whether the harms of VCSAM are represented in this reporting. These findings should prove valuable across policymakers, criminal justice departments, media, and psychology areas.

## Method

To attain the most up-to-date information, the search spanned 1 January 2019 to 23 September 2022 and resulted in 160 articles. The ProQuest Database was used, and a broad keyword search was employed across newspaper articles: (“virtual child” OR “cartoon” OR “deepfake” OR “digitally altered”) AND (“child exploitation material” OR “child sexual abuse material” OR “child abuse material” OR “child pornography” OR “imagery abuse”). A total of 135 articles were removed because they did not fit the inclusion criteria. The inclusion criteria of the newspaper articles were to: (1) discuss a specific VCSAM offender rather than the general topic of VCSAM offending; (2) be published in English; and (3) be published in a W country. Articles were still included if the offender engaged in both CSAM and VCSAM. Most of the 135 articles that were removed, many of which were duplicates, did not refer to a specific VCSAM case (i.e., only offered a broad discussion on VCSAM) or only mentioned a CSAM case in which the article did not include VCSAM. The final sample comprised 25 newspaper articles. A second researcher confirmed that the final sample met all inclusion criteria. It is important to note that while this form of data (periodical broadsheet newspapers) is more reliable than other forms of data (e.g., tabloid magazines), it is not as reliable as other types of data, such as forensic reports or legal transcripts. However, with the dearth of available data given VCSAM is such an emerging issue, the current data fills a clear stopgap until future research can use additional types of sources to support the current findings.

### Analytical Approach

The current study employed a qualitative content analysis. This method was selected over a quantitative approach as it allowed for reflexivity across data coding, analysis, and interpretation that was analytic and systematic, as opposed to being rigid (Altheide, 1987). This method allows one to understand the meaning communicated and is underpinned by constant discovery and comparisons and is mindful that new categories can emerge throughout the study (Altheide, 1987). This qualitative approach is “oriented to check, supplement, and supplant prior theoretical claims”. Given research has found some disconnect between the perceived harmfulness of VCSAM and legislative definitions and that such perceptions are possibly influenced by public debates (Prichard et al. 2015), it is, therefore, beneficial to explore whether media reports of VCSAM cases downplay the harms. This method is also considered to provide valuable descriptive information. The use of qualitative content analysis has already been recognized as an effective tool for attaining insights into how CSAM offenders are represented in the print media (Christensen & Pollard, 2022).

## Results

The results are presented in two parts: (1) case descriptives and (2) qualitative content analysis. The case descriptives offer insights into the characteristics of VCSAM offenders. The qualitative aspect provides a depiction of whether the harms of VCSAM are represented in this reporting to the public in the media.

### Case Descriptives

While most cases occurred in 2019 (32%), some cases stemmed back over a decade (minimum = 2008; maximum = 2021).^1^ Most newspaper articles reported crimes in Australia (48%) followed by the United Kingdom (24%), the United States (16%), and Canada (12%). There were 26 offenders across the 24 articles. All cases involved both VCSAM and CSAM, except for seven cases that were solely VCSAM. Case descriptives explored offender gender, offender age, mental health, how the material was discovered, number and type of charges, victim age and victim gender, types of cases, and the number of files, along with the plea, sentencing, and criminal history.

All offenders were male, except for one case that involved two offenders: one male and one female. The average age at offense onset was 32.81 years (minimum = 17 years; maximum = 69 years). 2 The average age at sentencing was 39.71 years (minimum = 19 years; maximum = 73 years).^3^ There was little mention of substance issues or mental health for most offenders. Only one article indicated the offender having alcohol and narcotic issues, and 20% of articles indicated some form of mental health issues or developmental disorders, including autism spectrum disorder, depressed mood disorder, pedophilic disorder, compulsive personality disorder, and voyeuristic disorder. Most articles did not document the offender’s employment. Of known cases, the offenders worked in the technology field, security, teacher aide, or were caregivers.

In terms of how the offending was discovered, of known cases, offenders were discovered using various intelligence. This included a report from Microsoft, police searches of homes, authorities becoming aware of IP addresses accessing CSAM, and acting on information from the public. For example, an attentive teacher noticed a phone was propped up in the changing areas of the children’s toilets. In another instance, a stranger located a lost phone with the material on it. The offending was also discovered from monitoring software that identified the material. In this instance, the offender had previously been convicted of sexual abuse on a young child and had monitoring software set up on his devices by authorities to prohibit visiting suspicious sites. This then led to the search of the offender’s vehicle, in which the offender’s probation officer discovered the offender to have an additional laptop that had illegal material on it. Offenders went to various lengths to avoid detections, with one offender utilizing multiple virtual private networks (VPNs).

The average amount of charges per offender was 5.74 (minimum = 0 charges; maximum = 35 charges). The case that did not result in a charge involved a 17-year-old male who received the cartoon images on his phone from a friend as a “joke”. Most cases had possession charges (84%), followed by making/recording charges (32%), access charges (20%), and distributing charges (16%).^4^ Almost half of the cases resulted in additional charges that were non-CSAM related. Most of these additional charges were sexual offenses, including sexual assault, sexual penetration of a child, and rape. Other offenses related to a bond breach, not complying with reporting obligations, and unlawful possession of a weapon.

Cases mostly involved pre-pubescent children in the material (86%), including toddlers. Only 14% of cases solely involved pubescent children. While the age depicted was often unspecified, the youngest characters were depicted as babies. Most children in the material appeared to be female (75% of cases). Few articles distinguished between the number of VCSAM images/videos and the number of CSAM images/videos, likely due to the sheer amount of the material. Across both types of material, there was an average of 10,354 images/videos (minimum = 2 images/videos; maximum 100,000 images/videos). Most of the VCSAM material was cartoons, anime, or computer-generated files. Some of the cartoons included internationally known cartoon characters. VCSAM was also represented in comics, fictional stories, GIFS, memes, and digitally altered children.

Most cases (88%) had been prosecuted.^5^ Of these cases, all offenders pleaded guilty, 73% were sentenced, 23% did not receive a sentence, and less than 5% had not yet been sentenced. Regarding sentence type, almost 56% of sentenced offenders received a sentence to be served in prison. The period of imprisonment ranged from 10 months to 27 years (mean = 3.99 years). The remaining sentenced cases concerned suspended sentences, a good behavior order, a conditional sentence to be served in the community, rehabilitation requirements, and a fine. Almost one quarter (24%) had a prior conviction, with all these prior convictions related to sexual offenses against children, including child luring, engaging in sexual activity with children, and the possession of CSAM.

### Themes

Four themes, and one sub-theme, emerged in the qualitative content analysis. The four themes entailed: (1) VCSAM is a form of CSAM, (2) potential for escalation in offending from viewing VCSAM, (3) offenders have preferences in specific types of VCSAM (with the sub-theme: written stories and documents are VCSAM too), and (4) offenders “didn’t know” the material was illegal. Each of these themes is discussed below.

#### VCSAM is a form of CSAM

Several articles drew from the voices of authorities and highlighted their messages that VCSAM *is* a form of CSAM, signifying that this type of material can still result in harm. Several cases included messages from judicial officers. For example, in one case, while the judicial officer indicated that drawn images and anime are considered to be less serious compared with pictures of real children, it was still considered to be “of great concern”. In fact, this judicial officer highlighted the “serious risks” of this material, having the potential to “degrade and incite”. In a different case, the judicial officer highlighted the reasonable “public concern” about any type of offense in relation to possessing CSAM and that regardless of whether the children were real or anime, protecting children is of “paramount importance” in society. Another judicial officer highlighted those individuals accessing this material “creates a market” and that this material can still involve the suffering and degradation of children. Another case drew from the voice of law enforcement, with VCSAM charges serving as “a reminder” to society that cartoon/animated images are illegal and can result in serious charges.

#### Potential for Escalation in Offending from Viewing VCSAM

The point of escalation in offending was also highlighted across a diverse group throughout the articles. For example, in one article, the director of a child sexual abuse survivor support center voiced the “very real” link between pursuing sexual abuse after viewing such images, irrespective of images being cartoons or involving real victims, arguing the sexualization process is alike. A prosecutor in a different case indicated that sexual offending might be spurred on by the normalization of the cartoon material and through the ability to use it to groom children. In a different case, in which the offender had over 30,000 images, and over 600 videos, all computer-generated or cartoon/anime files, the offender himself said that he was concerned about the effect the material had on him, and he could see himself engaging in worse offending as a result, including perpetrating similar offenses on children.

#### Offenders have Preferences in Specific Types of VCSAM

Offenders appeared to have an interest in certain types of VCSAM as opposed to a variety of styles.^6^ For example, in one case, the offender solely possessed memes. In a different case, the offender had GIFs. In this instance, the offender had made these GIFs himself by using photographs of girls in his local area and making her head move in the GIFs, performing sexual acts on men. Another offender had anime cartoon images, while other offenders had well-known children’s cartoon characters from The Simpsons that had been altered to engage in sex acts, and a sexualized image of Astro Boy. In another case, the offender had transposed the face of a child onto a sexually explicit photo involving another real child, making the image appear as if the transposed child was being abused. Alarmingly, the child’s face the offender used was that of someone he had sexually abused in the past. In comparison to these cases, there was one case in which an offender possessed a range of cartoon strips, comics, and computer-generated images. This range of VCSAM, as opposed to having one set type, was likely due to the fact this offender had over 21,000 files in total.

A sub-theme within this theme emerged – written stories and documents are VCSAM too – with a number of offenders possessing written stories and documents. For example, in one case, the stories included the rape and torture of children depicted between the ages of 10 and 16. In a different case, a man was charged for possessing a fictional story about a toddler being raped in addition to charges for contact offending, including the sexual penetration of a child. Another offender possessed a written letter about a father sexually penetrating his 12-year-old daughter. Further to written stories, offenders had other types of written documents. For example, one offender possessed several “pedophile manuals”. These manuals detailed how to groom, abuse, and “hide” children. In this case, the offender also had a sexual interest in animals, having engaged in bestiality. He also disclosed to his psychologist that he was a pedophile and wore a chastity belt to avoid abusing children, which was located during the police search of his home. In a different case, an offender had a written document that outlined sexual offenses being perpetrated against children. Regardless of the type of material, some articles included the relevant legislation highlighting to the readers the illegality of the material, including written material.

#### Offenders “didn’t know” the Material was Illegal or Downplayed the Offending

The final theme to emerge was the suggestion by offenders that they did not realize the material was illegal or offered some form of downplay. This was typically met with denunciation by judicial officers. In one article, the offender commenced using the material by “rationalizing” that the material was not really illegal. In a different case in which the offender created and ran servers on the dark net, which provided access to the material, the offender had complained on a chatroom that one of his servers was disconnected by an internet service provider due to a “fake” CSAM complaint. When one chatroom member highlighted that the material can be considered illegal, the offender argued that the federal authorities have more important things to do than “go after cartoons”, downplaying the gravity of the material. In a separate article, the offender suggested they did not think the material involved underage females, however, this was met with the judicial officer highlighting it was “difficult to understand” how the offender did not see this. In a different case, one judicial officer reprimanded the offender, emphasizing it was troubling that the offender suggested he did not have any sexual interest in the material. This judicial officer made it clear that he did not believe the offender. Finally, some barristers argued that their client had “blurred the line” in regard to their fetishes or was socially awkward and used the sexual activity to “connect with people”, appearing to downplay the offending.

## Discussion

The aim of this exploratory study was to: (1) identify the characteristics of VCSAM offenders and (2) explore whether the harms of VCSAM are represented in this reporting. This research is timely, given the dearth of literature on VCSAM offenders and the clear disconnect between the public’s perceived harmfulness of VCSAM and legislative definitions. The dearth of literature is particularly troubling when law enforcement and child welfare experts have flagged concerns about the increasing issue of VCSAM for over a decade (Northern Ireland Office, 2007). This section explores the characteristics of VCSAM offenders, followed by the depiction of whether the harms of VCSAM are represented in this reporting to the public. The four themes concerning the reporting of harms were: (1) VCSAM is a form of CSAM, (2) potential for escalation in offending from viewing VCSAM, (3) offenders have preferences in specific types of VCSAM (with the sub-theme: written stories and documents are VCSAM too), and (4) offenders “didn’t know” the material was illegal. Limitations, implications, and ideas for future research are also discussed.

### Characteristics of VCSAM Offenders

Several key findings emerged that pertained to offender gender, offender age, mental health, how the material was discovered, number and type of charges, victim age and victim gender, types of cases, and the number of files, along with the plea, sentencing, and criminal history. First, in terms of offender gender, it was surprising to find that all offenders were male (except for one case that involved one male and one female) when females can play a lead role in CSAM offending (Bickart et al., 2019). In fact, recent research has found the media reports on female CSAM cases in a way that portrays them as actively involved in offending as opposed to passive offenders (First Author, under review). While the current dataset is limited, the current findings suggest that females who engage in CSAM offending *might* be less interested in VCSAM offending compared with their male counterparts. Future research could explore a larger group of VCSAM offenders to see whether this is the case and, if so, the reasons for the gendered difference.

As for offender age (39.71 years at sentencing), this was consistent with previous research on CSAM offenders (40.61 years at sentencing; Christensen & Tsagaris 2020). Few offenders were reported to have mental vulnerabilities. It was not surprising to find the array in how the offending was discovered, involving intelligence agencies through to reports from members of the public, consistent with previous CSAM research (Christensen & Pollard, 2022). Such reporting on how cases are discovered is valuable in potentially deterring this offending when drawing from the rational choice perspective (Cornish & Clarke, 1986) argue that offenders engage in criminal behavior when the benefits prevail over the potential costs. If the detection of VCSAM through various types of intelligence is being reported on, the salience of these cases may deter some potential offenders from offending.

One interesting finding was the high number of charges (an average of 5.74 charges). Previous research has found a lower average, for example, 3.31 counts (Christensen & Tsagaris, 2020). One reason for this finding could be that the current study utilizes media reports, and thus more ‘newsworthy’ cases are reported on. However, similar research on the reporting of CSAM offenders in the media indicates low averages too (on average 1.61 charges; Christensen & Pollard 2022) which rebuts this suggestion. Another reason for this finding might be that with the consistent growth in technology and new platforms, offenders are committing more crimes and are therefore being charged at a higher rate.

Most cases involved possession offenses, which was consistent with previous research (Christensen & Pollard, 2022; Christensen & Tsagaris, 2020; Jung & Stein, 2012). The majority of cases involved pre-pubescent children in the material (only 14% of cases solely involved pubescent children), and most children were female. While the finding on age could be attributable to these cases being ‘newsworthy’, the current finding is consistent with the literature that has reported an ongoing decrease in victim age (European Commission, 2015). The finding regarding the preference toward female children is also consistent with recent literature (Christensen & Pollard, 2022). Overall, offenders had a vast number of files (an average of 10,354 images/videos). It was, therefore, not surprising to find that most cases involved both VCSAM and CSAM, except for seven cases that were solely VCSAM. As discussed below, there was an array of VCSAM material, including cartoon, anime, or computer-generated files, comics, fictional stories, GIFS, memes, and digitally altered children.

The high guilty plea was consistent with previous research (Christensen & Pollard, 2022; Christensen & Tsagaris, 2020; Jung & Stein, 2012). The finding that most offenders received a sentence to be served in prison was also consistent with prior literature (Christensen & Tsagaris, 2020; Jung & Stein, 2012). This was a positive finding as this reporting in the media could deter potential offenders from engaging in offending. In line with the rational choice perspective (Cornish & Clarke, 1986), some offenders might perceive that the risks are too costly (i.e., time in prison), outweighing the benefits of engaging with the material (e.g., a masturbatory aide). Unlike previous research on CSAM offenders (e.g., Babchishin et al., 2011; Christensen & Tsagaris, 2020; McCarthy, 2010), a sizeable proportion of cases (24%) had a prior conviction; all of which related to sexual offenses against children and the possession of CSAM. One reason for this finding could be the media reporting on more ‘newsworthy’ cases. Although, few of the CSAM offenders in Christensen and Pollard’s (2022) media study had a criminal history. Regardless of the reason, the reporting of offenders with criminal histories is positive as it may act as a form of deterrence by sending the message to *repeat offenders* that recidivists are detected by authorities.

### Themes

Four themes, and one sub-theme, emerged in the qualitative content analysis. The four themes were: (1) VCSAM is a form of CSAM, (2) potential for escalation in offending from viewing VCSAM, (3) offenders have preferences in specific types of VCSAM (with the sub-theme: written stories and documents are VCSAM too), and (4) offenders “didn’t know” the material was illegal. The theme ‘VCSAM is a form of CSAM’ was a positive finding. The articles voiced the concerns of authorities, often drawing from the sentencing remarks made by judicial officers, highlighting the material is still harmful despite the images being drawn. Sentencing remarks are considered to be a useful tool for both crime prevention and informing the public about the wrongs of CSAM (Hunn et al., 2018). Given that CSAM offenders are found to engage in self-distancing in which they downplay their offending and accountability (Winder & Gough, 2010), it is not unreasonable to suggest that individuals who engage in VCSAM offending might engage in even greater self-distancing due to the nature of the, often fictional, material. In turn, these authoritative voices providing the message to the public that VCSAM *is* a form of CSAM could be pivotal in the primary prevention of VCSAM. While the findings of this research suggest judicial officers condemn VCSAM, it also highlighted some disparate sentencing among similar offenses, with one article scrutinizing the empathy of the judiciary towards the offender. One area for future research is the exploration of judicial officers’ perceptions in relation to the harms of VCSAM.

Another theme to emerge was the ‘potential for escalation in offending from viewing VCSAM’. The articles reported on the link between engaging in the material and contact offending, along with the normalization of the material, and the ability to use the material to groom children. This was another constructive finding as it is consistent with the literature that argues VCSAM can serve as a gateway to contact offending, acting as a progressive addiction (Maras & Shapiro, 2017; Christensen et al., 2021) provide the hypothetical example that one may begin using VCSAM material to masturbate and, become desensitized over time, escalating to CSAM material, before progressing to contact offending to reach the level of gratification he or she felt when they initially engaged with VCSAM material. It was also interesting to find in the current study that one offender themselves even acknowledged the potential for escalating to worse offending. This reporting highlights to the public the potential impacts of the offending. One idea for future research is to explore the offending trajectories of VCSAM offenders to understand this population further, with a focus on identifying offending patterns, including the escalation of offending. Interviews with VCSAM offenders could provide very useful insights.

It was particularly interesting identifying the theme, ‘offenders have preferences in specific types of VCSAM’ as opposed to being engaged in a diversity of the material. Preferences included cartoons (including well-known cartoon characters), anime, GIFS, memes, and comics. Alarmingly, one offender morphed a child’s face (whom he had previously abused) on a sexually explicit image. One reason for this finding could be that similar to individuals having preferences for sub-types of pornography (e.g., female insatiability, non-consenting adult pornography, erotic, etc.; Bogaert 2001), individuals could have preferences for certain types of VCSAM. Future research needs to test this finding on a larger sample of VCSAM offenders. If a similar trend emerges, one idea would be to explore the mediating mechanisms (e.g., cognitive and emotional components) between individual differences (e.g., personality) and preferences for VCSAM (e.g., cartoon, anime, written, etc.). Such findings could potentially assist in tailoring prevention and intervention efforts.

Regarding the sub-theme ‘written stories and documents are VCSAM too’, it was interesting to find several offenders had written stories and documents, such as fictional stories and “pedophile manuals”. This group of offenders appeared to be quite heterogeneous in their characteristics. While the argument could be made that some offenders might access this material in the hopes of being less likely to be detected by authorities due to it being in written format, this proposition does not hold for the current cases as all offenders had additionally accessed visual VCSAM material and/or CSAM. Another reason for this finding could simply be that some individuals had preferences for this type of material. It is not irrational to argue that some members of the public might perceive written VCSAM documents as less harmful compared with visual VCSAM. In turn, the reporting of these cases could potentially educate the public about this type of offending, along with acting as a form of deterrence for potential offenders. In order to understand this group of individuals further, future research with VCSAM offenders could explore how they perceive the harms of the written material compared with the visual content and whether the use of the written material escalated to the use of visual content (or vice versa). This information could also assist with intervention and prevention initiatives. Regardless of the type of material, some articles included the relevant legislation highlighting to the readers the illegality of the material, including written material. The accessibility of the different types of VCSAM available online also highlights the potential for offenders to easily engage in escalation (i.e., resulting in further harmful behavior).

The final theme was ‘offenders “didn’t know” the material was illegal or downplayed the offending’. This finding was not surprising, given that previous research has found that downplay and denial was the most noted explanation for offending (Christensen & Tsagaris, 2020). The current finding is also consistent with Winder and Gough (2010), who found in their interviews with CSAM offenders that offenders engaged in ‘self-distancing’, minimizing their offenses. Fortunately, a number of the articles in the current study reported on the denunciation and reprimand offered by judicial officers, highlighting the harms. As outlined by Hunn et al. (2018), the judiciary can play an educative role and contribute to primary prevention through their authoritative explanations of the wrongfulness of offending, and the media is an important vehicle for disseminating the judiciary’s remarks. Individuals contemplating their first offense in VCSAM might see such articles and realize that no weight will be given to their argument that they ‘didn’t know’, for example, that cartoon material is illegal. While one cannot be certain individuals at risk of onset read such articles in the media, such messages offered by the judiciary cannot be overlooked in terms of a larger prevention context (Hunn et al., 2018). It would, however, be ideal at the end of such articles to link appropriate offender-oriented prevention referral services (e.g., *Stop It Now*! Helplines) for individuals who are concerned about their own behavior.

### Limitations

Despite the paper’s valuable contribution in identifying the characteristics of VCSAM offenders and exploring whether the harms of VCSAM are represented in the reporting of print media, a number of limitations are evident. First, one major limitation was the current study having a sample size of 25 newspaper articles. While the sample could have been larger had the timeframe been extended before 2019, the researchers wanted to capture the most up-to-date data, particularly given the increasing speed of technological enhancements impacting VCSAM and the fact that VCSAM is still prompting conjecture in the present day as to whether such content is harmful (Moritz et al., 2022). Second, it must be acknowledged that the quantitative data was calculated based on the known cases. The authors acknowledge a limitation of using this type of data is the amount of missing information. However, given the absolute dearth of literature that exists, the data has still offered valuable insights. Further, ideas for future research have been proposed to explore some of these gaps. Third, the current study’s sample did not involve solely VCSAM-only cases (except for seven cases). As mentioned, it was not surprising to find that most cases involved both VCSAM and CSAM, given the vast number of files offenders had. While the current study was restricted by this sample, future research could explore differences in characteristics and motivations across CSAM offenders, CSAM and VCSAM offenders, and VCSAM-only offenders through, for example, interviews across these offender groups.

## Conclusion

The aim of this exploratory study was twofold and sought to: (1) identify the characteristics of VCSAM offenders and (2) explore whether the harms of VCSAM are represented in this reporting. Several findings emerged that pertained to offender gender, offender age, mental health, how the material was discovered, number and type of charges, victim age and victim gender, types of cases, and the number of files, along with the plea, sentencing, and criminal history. With the dearth of literature on VCSAM offenders, these findings offer very valuable insights into the characteristics of VCSAM offenders. Four themes emerged in the current study regarding the representation of the harms of VCSAM are: (1) VCSAM is a form of CSAM, (2) potential for escalation in offending from viewing VCSAM, (3) offenders have preferences in specific types of VCSAM (with the sub-theme: written stories and documents are VCSAM too), and (4) offenders “didn’t know” the material was illegal. Overall, these themes were positive to find; the articles signify the harms of this offending and may broadly educate the public about VCSAM offending. Further, such articles may potentially assist with preventing both first-time and recidivist offenders, when applying a rational choice theory lens. Future research needs to test the current findings on larger samples of VCSAM offenders, with a focus on VCSAM preferences, to assist in tailoring prevention and intervention efforts.

## Data Availability

Not applicable.

## Footnotes

1 It must be acknowledged that the quantitative data in the Results has been calculated based on the known cases. The authors acknowledge a limitation of using this type of data is the amount of missing information. In instances where the offending stretched over several years, the year the offending commenced was used in the current calculation.

2 This figure removes cases where only rough ages were provided (e.g., ‘in 50’s’). Some ages were calculated based on dates. As aforementioned, this data has been calculated based on the known cases.

3 Similar to age at offence, this figure removes cases where only rough ages were provided.

4 This value adds up to over 100% as many cases had several different types of charges.

5 Two cases did not result in prosecution, and one case did not specify the outcome.

6 This was an overall finding. It must be acknowledged that bigger quantities of CSAM and VCSAM make it more difficult to identify the specific types of VCSAM.
